# Central Sensitization and Psychological State Distinguishing Complex Regional Pain Syndrome from Other Chronic Limb Pain Conditions: A Cluster Analysis Model

**DOI:** 10.3390/biomedicines11010089

**Published:** 2022-12-29

**Authors:** Hana Karpin, Jean-Jacques Vatine, Yishai Bachar Kirshenboim, Aurelia Markezana, Irit Weissman-Fogel

**Affiliations:** 1 Physical Therapy Department, Faculty of Social Welfare and Health Sciences, University of Haifa, Haifa 3498838, Israel; 2 Reuth Rehabilitation Hospital, Tel Aviv 6772829, Israel; 3 Physical Medicine and Rehabilitation Department, Sackler Faculty of Medicine, Tel Aviv University, Tel Aviv 6997801, Israel; 4 Department of Occupational Therapy, School of Health Professions, Sackler Faculty of Medicine, Tel Aviv University, Tel Aviv 6997801, Israel; 5 Goldyne Savad Institute of Gene Therapy, Hadassah Hebrew University Medical Center, Jerusalem 91120, Israel

**Keywords:** complex regional pain syndrome, chronic limb pain, nociplastic pain, psychological state, cluster analysis, CRPS clinical phenotypes

## Abstract

Complex regional pain syndrome (CRPS) taxonomy has been updated with reported subtypes and is defined as primary pain alongside other chronic limb pain (CLP) conditions. We aimed at identifying CRPS clinical phenotypes that distinguish CRPS from other CLP conditions. Cluster analysis was carried out to classify 61 chronic CRPS and 31 CLP patients based on evoked pain (intensity of hyperalgesia and dynamic allodynia, allodynia area, and after-sensation) and psychological (depression, kinesiophobia, mental distress, and depersonalization) measures. Pro-inflammatory cytokine IL-6 and TNF-α serum levels were measured. Three cluster groups were created: ‘CRPS’ (78.7% CRPS; 6.5% CLP); ‘CLP’ (64.5% CLP; 4.9% CRPS), and ‘Mixed’ (16.4% CRPS; 29% CLP). The groups differed in all measures, predominantly in allodynia and hyperalgesia (*p* < 0.001, η² > 0.58). ‘CRPS’ demonstrated higher psychological and evoked pain measures vs. ‘CLP’. ‘Mixed’ exhibited similarities to ‘CRPS’ in psychological profile and to ‘CLP’ in evoked pain measures. The serum level of TNF-αwas higher in the ‘CRPS’ vs. ‘CLP’ (*p* < 0.001) groups. In conclusion, pain hypersensitivity reflecting nociplastic pain mechanisms and psychological state measures created different clinical phenotypes of CRPS and possible CRPS subtypes, which distinguishes them from other CLP conditions, with the pro-inflammatory TNF-α cytokine as an additional potential biomarker.

## 1. Introduction

Complex regional pain syndrome (CRPS) is a multifaceted pain disorder that mainly emerges after limb trauma or a lesion in the peripheral nervous system. The syndrome is comprised of CRPS-Type I (without nerve damage) and CRPS-Type II (with major nerve damage) subtypes, although their clinical presentation is similar [1]. Typical features include continuing pain (disproportionate to any inciting event or underlying pathology), and sensory, vasomotor, sudomotor, motor, and trophic changes [2,3]. The syndrome is diagnosed according to the Budapest Criteria, a decision rules method based on objective clinical signs and reported self-subjective symptoms [4]. Epidemiologically, the syndrome’s incidence rates range between 5.6 [5], 26.2 [6], and 29 [7] per 10,000 person-years in the USA, Europe, and Korea, respectively.

The severity of the syndrome may be determined by the CRPS Severity Score (CSS), which reflects features of CRPS included in the Budapest Criteria [4] CRPS was recently defined as a Chronic Primary Pain (CPP) disorder, a pain condition in its own right which is not better accounted for by another disease. The pain persists for over three months and is associated with significant emotional distress and/or functional disability [8]. Mechanistically, CPP is viewed as nociplastic pain that is maintained by abnormal central processes, i.e., hypersensitivity of pain transmitting pathways (i.e., central sensitization) and/or an inefficient endogenous pain inhibition process. These are manifested as hyperpathia, hyperalgesia, and allodynia in response to evoked pain [9]. Nociplastic pain was accepted by the IASP as a third mechanistic pain descriptor in addition to neuropathic and nociceptive pain [9].

The literature suggests that CRPS is a heterogeneous syndrome based on different pathophysiological mechanisms [10,11] including central sensitization, inflammation, immune alterations, brain changes, genetic predisposition, and psychological state [12,13]. Cluster analysis procedures that were performed to detect different CRPS subtypes have yielded various classifications: ‘warm’ (inflammatory) vs. ‘cold’ (chronic) [11] and ‘central’ (maladaptive sensory-motor processing) vs. ‘peripheral’ (inflammatory signs) subtypes [10]. Additional classification is based on the serum level of TNF-α; in the high-level TNF-α group, levels of TNF-α were shown to be correlated with greater disease severity and longer disease duration [14]. Other classifications were focused on the syndrome’s cognitive, perceptual, or emotional features. For example, different subtypes of neuropsychological function in CRPS patients (i.e., normal performance, executive difficulties, and global cognitive impairment) [15] or ‘motor’ vs. ‘cognitive’ neglect reflecting different phenotypes of body perception disturbances [16]. However, research aimed at subgrouping CRPS based on clinical features has failed to support the traditional sequential staging of CRPS mainly because the pain duration was similar across the subgroups [1,14].

Recently, the CRPS taxonomy was updated [17] with newly reported subtypes and a clarified diagnosis procedure. Specifically, the updated taxonomy includes two CRPS subtypes: (1) ‘CRPS Not Otherwise Specified’ meaning patients who display insufficient features of CRPS that are required for formal diagnosis (based on the Budapest Criteria) with no other diagnosis to better explain their clinical state, and (2) ‘CRPS with Remission of Some Features’ (CRPS RoSF) meaning patients that were previously diagnosed with CRPS but then display insufficient signs and symptoms to fully meet the diagnostic criteria [17]. However, it is still unclear whether these subtypes are distinct subtypes with unique clinical features or are part of the sequential stages of the syndrome reflecting recovery processes [17]. Moreover, to improve the therapeutic outcome of CRPS patients, it is essential not only to identify subgroups within CRPS that probably reflect different mechanisms but also to differentiate CRPS from other chronic limb pain (CLP) conditions. 

The diagnostical definition of CLP has been changed recently, including the differentiation between primary chronic limb pain (which is considered a disease on its own [18,19] and secondary chronic limb pain when the pain is defined as a symptom [19,20]. Although these clarifications give more precision to the diagnostic process, many chronic pain conditions involve a combination of pain mechanisms [9,21], which makes this process harder and accordingly requires more clinical clarifications.

A previous study that explored measures that differentiate CRPS patients from CLP patients found that spontaneous pain, pinprick hyperalgesia, and dynamic allodynia were more prominent in CRPS patients. This suggests that augmented pain in CRPS [3] can be attributed at least in part to central sensitization. In another study, CRPS and CLP patients were compared based on a broad clinical but limited psychological battery. Two discriminating factors were identified: (i) clinical pain based on self-reporting and evoked pain (i.e., pinprick and pressure pain sensitivity), and (ii) psychological measures (i.e., anxiety and depression). CRPS demonstrated higher intensity in both factors [22]. Additional psychological factors including catastrophization [23,24], kinesiophobia [25,26], and somatization characterize CRPS and other CLP conditions [24]. Interestingly, depersonalization is the only measure that distinguishes CRPS from other chronic pain groups (i.e., CLP and migraine) [24]. 

An interesting potential phenotype of CRPS, namely Body Perception Disturbances (BPD), which is measured by limb neglect, was compared between CRPS and CLP patients. Results showed that neglect scores were higher in CRPS patients and that they were correlated with psychological factors. Nonetheless, the neglect scores were associated with clinical pain intensity only in CLP patients, indicating a different role for BPD in various CLP disorders [24].

Taken together, the evidence suggests a combination of factors, specifically those reflecting pain hypersensitivity and psychological distress distinguishes CRPS from CLP. Therefore, the current study’s aims were: (1) to identify phenotypes that distinguish CRPS from other CLP based on comprehensive evoked pain and psychological measures, and (2) to explore the validity of these measures and their usage as a novel, clinically oriented classification method. The main findings show that nociplastic pain and the level of psychological distress can distinguish between CLP conditions; the CRPS group has a unique psychological and pain profile derived from central sensitization and central neuro-inflammation processes.

## 2. Materials and Methods

### 2.1. Patients 

This was a cross-sectional observational study comprised of two patient groups: a research group and a control group. The inclusion criteria for the research group were: (1) subjects aged > 18; (2) diagnosed with CRPS type 1 or 2 in the upper or lower limb, according to a medical evaluation of a pain specialist physician and based on the Budapest clinical criteria; and (3) pain that has persisted > 4 months since the primary injury. The exclusion criteria were: (1) bilateral CRPS; (2) primary psychiatric diagnosis of depression, anxiety, or post-traumatic stress disorder; (3) a different pain syndrome causing a major pain; (4) disorders of the central nervous system (epilepsy, intracranial injury, stroke, Parkinson’s disease, multiple sclerosis), or other diseases with sensory or inflammatory components; (5) pregnant or nursing women; (6) severe visual deficiency; (7) intellectual disability; and (8) insufficient proficiency in spoken Hebrew. 

The control group was comprised of subjects aged> 18 who were diagnosed with chronic secondary musculoskeletal pain [20] according to a medical evaluation by a pain specialist physician. The pain in this group was from a nociceptive origin and associated with traumatic structural changes in the musculoskeletal system and some of the subjects in this group also had a nerve injury. All subjects in this group did not reach the CRPS criteria [27]. Both patient groups were referred to the pain rehabilitation units at Reuth Rehabilitation Hospital (Tel-Aviv, Israel) and were voluntarily recruited during their rehabilitation course from June 2018 to January 2021. The study was approved by the Institutional Review Board (IRB) of Reuth Rehabilitation Center (2017-14) and by the IRB of the University of Haifa (Haifa, Israel, 135/18; File 1822). Participants signed written informed consent forms before inclusion in the study.

### 2.2. Disease Severity Measures

#### 2.2.1. CRPS Severity Score

The CSS [4,27] is an index consisting of 16 signs and symptoms, including sensory, vasomotor, sudomotor/edema, and motor/trophic criteria. The index is scored based on the presence/absence (coded 1/0) of signs and symptoms. A higher CSS score indicates a greater extent of CRPS symptoms. 

#### 2.2.2. Short-Form McGill Pain Questionnaire (MPQ-SF)

The MPQ-SF [28] is a self-report questionnaire that assesses the multi-dimensional aspects of current clinical pain. The questionnaire includes 11 sensory descriptions and four emotional descriptions (on a 0–3 scale; no, mild, moderate, and strong, respectively). The total score ranges from 0 to 45 points; a higher score represents a more intense pain experience. The MPQ includes two measures of pain intensity: The Visual Analogue Scale (VAS) with the two ends denoting ‘no pain’ and ‘maximal pain imaginable’, and a verbal pain bar ranging from 0 ‘no pain at all’ to 5 ‘very strong pain’. We used the validated Hebrew version of the SF-MPQ [29].

### 2.3. Psychophysical Measures

The psychophysical tests were performed on the dorsal aspect of the involved hand or foot, in the area defined as having ‘secondary hyperalgesia’ (i.e., increased pain sensitivity in non-injured skin surrounding a site of tissue damage [30]. The rationale was to explore and quantify central processes and to prevent a ceiling effect (i.e., unbearable pain) in case the stimulus was placed in the center of the primary hyperalgesia area. In cases where allodynia or hyperalgesia was not identified, the tests were taken adjacent to the injury site. If this area was scarred, the tests were performed on the nearest intact skin area.

#### 2.3.1. Thermal and Pain Thresholds 

This set of tests was performed by an occupational therapist according to the standardized DFNS (German Research Network on Neuropathic Pain) protocol [31]. The threshold tests included cold detection threshold (CDT), warm detection threshold (WDT), cold pain threshold (CPT), and heat pain threshold (HPT) that were performed on the dorsal aspects of the hand or the feet of both sides of the body, depending on the affected extremity [22], with the unaffected side tested first. The thermal tests were applied before the pain threshold tests. The threshold tests were performed by the Thermal Sensory Analyzer (TSA) system (Medoc, Ltd., Ramat Yishai, Israel) using the 3 × 3 cm contact thermode that warms and cools using a temperature range from 0 °C to a safety limit of 50 °C. The baseline temperature was 32 °C and the thermode heat and cool rate was 1 °C/s. For the WDT and CDT, the participant was asked to press a computer mouse button when warm and cold sensations were felt. For the HPT and CPT, they were asked to detect the moment that warm and cold sensations became painful and to press the button. Each test was performed three times and averaged. The mean value of each test was Z transformed, based on the following equation, according to the standardized and published instructions [31].

Z score = (patient mean value) − (mean value of the published reference) ÷ SD of the published reference.

The z scores were adjusted as follows: z score > 0 indicated high sensitivity and z score < 0 indicated low sensitivity; z score > +1 indicated somatosensory gain and z score < −1 indicated somatosensory loss; z score > ±1.96 indicated pathologic changes in somato-sensory function [10].

#### 2.3.2. Mechanical Hyperalgesia Intensity 

The intensity of supra-threshold mechanical stimuli was assessed using a pinprick stimulator at 256 mN (MRC Systems Pin Prick Stimulator, Heidelberg, Germany). The participant was asked to rate the pain intensity using the 0–10 Numerical Rating Scale (NRS; 0 denoted ‘no pain’ and 10 denoted ‘maximal pain imaginable’). 

#### 2.3.3. Static Mechanical Allodynia Intensity 

The intensity of static allodynia was assessed using a cotton stick. The stimulus was applied once perpendicular to the skin, for two seconds, with a pressure force that was less than 15 gr. [32]. The participants were asked to rate the pain intensity using the 0–10 NRS. 

#### 2.3.4. Dynamic Mechanical Allodynia Intensity 

The intensity of dynamic allodynia was assessed using a cotton stick. The stimulus was applied once perpendicular to the skin, while the tester moved over a five cm skin area for two seconds with a pressure force that was less than 15 gr [32]. The participants were asked to rate the pain intensity using the 0–10 NRS. 

#### 2.3.5. Dynamic Allodynia Area 

We used a novel method developed for the current study to quantify the allodynia area [33]. The method protocol consisted of (i) identification and marking of the allodynia area using a cotton swab and makeup pencil; (ii) measurement of this area length with a cm tape; and (iii) calculation of the allodynia area by using the Lund and Browder chart for estimation of burned areas as a percentage of the total skin area (i.e., % fragment) [33,34], according to the following equation:

Allodynia area (%) = Length of allodynia area ÷ Length of body fragment × % fragment.

The calculated score represents the percentage of allodynia area relative to body surface. 

#### 2.3.6. Aftersensation Intensity 

Continued pain beyond the noxious stimulus presentation was recorded using the VAS immediately after the completion of the dynamic allodynia area test.

### 2.4. Psychological Self-Reported Measures

#### 2.4.1. Pain Catastrophizing Scale (PCS) [35] 

The PCS is a questionnaire that was developed to test an exaggerated negative mental set during actual or anticipated painful experiences [35]. The PCS includes 13 items on a scale ranging from 0 ‘not at all’ to 4 ‘all the time’ that represent three aspects of catastrophic thoughts related to pain: rumination (4 items), magnification (3 items), and helplessness (6 items). The overall score ranges from 0 to 52. The higher the score, the more negative the attitude toward the pain. We used the validated Hebrew version of the PCS [36]. 

#### 2.4.2. Tampa Scale of Kinesiophobia (TSK) [37] 

The TSK is a questionnaire that was developed to test fear of movement, fear of physical activity, and fear avoidance. The TSK includes 17 items ranging from 1 (‘I do not agree’) to 4 (‘completely agree’). The overall score ranges from 17 to 68. The higher the score, the higher the level of kinesiophobia. We used the standard Hebrew version of the TSK [38].

#### 2.4.3. Beck Depression Inventory (BDI-II) [39] 

This rating inventory measures characteristic attitudes and symptoms of depression. The BDI-II includes 21 items (on a scale of 0–3). The overall score ranges from 0 to 63. Higher scores suggest a greater severity of depression. We used the validated Hebrew version of the BDI-II [40].

#### 2.4.4. Brief Symptom Inventory (BSI) [41]

The BSI evaluates mental distress expressed in nine dimensions: obsessive-compulsive, interpersonal sensitivity, depression, anxiety, hostility, somatization, phobic-anxiety, paranoid ideation, and psychoticism. The BSI consists of 53 items on a scale ranging from 0 (‘not at all’) to 4 (‘extremely’). It can be summed up based on an overall score (General Severity Index) calculated using the sum of the nine symptom dimension scores and dividing by the total number of items. The higher the score the higher the level of mental distress. We used the validated Hebrew version of the BSI [42].

#### 2.4.5. Cambridge Depersonalization Scale (CDS) [43] 

The CDS measures the frequency and duration of depersonalization symptoms during the past six months. The CDC includes 29 items coded by two scales—a frequency scale ranging from 0 (never) to 4 (always) and a duration scale ranging from 1 (seconds) to 6 (over a week). The final score ranges from 0 to 290 points; a score above 70 was determined to be clinically significant depersonalization. The CDS was Hebrew translated for the benefit of the study using the back translation method [44] by four stages: (i) the CDS was translated to Hebrew by a bilingual occupational therapist; (ii) the Hebrew version was retranslated to English by a different bilingual occupational therapist; (iii) the two versions were compared for concept equivalence; and (iv) the final version was synthesizing after a discussion between the two translators. The internal consistency of the Hebrew scale was high (Cronbach’s alpha= 0.94).

#### 2.4.6. The Bath Body Perception Disturbances Questionnaire (Bath-BPD) [45] 

The Bath-BPD is comprised of seven items covering different aspects related to the affected limb: a sense of ownership, limb position awareness, attention to the painful limb, feelings toward the limb, perceptual disparities in size, temperature, pressure, and weight, limb amputation desire, and a mental representation of the affected limb. The total score is calculated by summing the individual scores of the seven items ranging from 0 to 57 points. The higher the score, the greater the degree of disturbance [44]. We used the Hebrew version of the Bath-BPD that was translated using the Vallerand method [45]. The internal consistency of the Hebrew-translated questionnaire was similar to the original English version (Cronbach’s alpha = 0.63 and 0.66, respectively) [46].

### 2.5. Biological Measures

#### Pro-Inflammatory Cytokines 

The blood samples were drawn using standardized venipuncture on the participants’ non-affected arm; 2 mL plasma was extracted from each patient and collected into EDTA-coated (purple top) vacutainers. After clotting, the plasma was separated by centrifugation (1500 rpm, 10 min, at 4 °C), the serum was extracted and then centrifuged again (3000 rpm, 10 min at 4 °C), split into two 250 microliter aliquots and stored at −20 °C until assayed.

The serum levels of Tumor Necrosis Factor- α (TNF-α) and Interluekin-6 (IL-6) were measured by a human TNF-α immuno-assay (Quantikine HS ELISA HSTA00E, R&D Systems, Minneapolis, Minnesota) and a human IL-6 immuno-assay (Quantikine ELISA D6050 R&D Systems, Minneapolis, Minnesota), respectively. The kits were used according to the manufacturer’s instructions by a trained laboratory technician. 

All samples were tested in duplicate and data were obtained by the standard curve that was created using the recombinant standards and expressed as the average protein levels in pg/mL for each group. Measurements were performed on the Infinite F50 ELISA microplate reader (TECAN Ltd., Männedorf, Switzerland) together with the Magellan^TM^ reader control and data analysis software (TECAN Ltd. Switzerland).

### 2.6. Statistical Analysis

The required sample size was estimated a priori for the ANOVA procedure that tested the cluster model sensitivity. Using the G*power program [47], α = 0.05, statistical power of 85%, and effect size of 0.35, the calculation yielded a total sample size of *N* = 93 participants. The final sample comprised 92 participants (61 CRPS and 31 CLP patients). 

All statistical analyses were performed using SPSS version 27.0 for Windows. Data are presented as mean ± SD or as median (in the case of non-parametric analysis) for continuous variables and as count and percentage for categorical variables. Effect sizes are presented by partial eta squared [48]. The statistical significance was defined as a value of *p* ≤ 0.05.

A repeated measures ANOVA was performed to compare the effect of group membership (CRPS/CLP) and the tested side (affected vs. not affected) on the thermal and pain thresholds.

A cluster analysis was performed to classify the sample based on evoked pain and psychological measures, aiming to identify different clinical phenotypes within the study sample. Eight measures were selected for the final model (four evoked pain measures: dynamic allodynia intensity, mechanical hyperalgesia intensity, dynamic allodynia area, and after-sensation intensity; and four psychological measures: depression, mental distress, kinesiophobia, and de-personalization). Two measures: the intensity of static mechanical allodynia and the PCS questionnaire were excluded due to their strong positive correlation with dynamic mechanical allodynia intensity and kinesiophobia, respectively (*r* > 0.57, *p* < 0.001). 

A statistical procedure of Two-Step Cluster Analysis was chosen due to its ability to ensure that one variable does not dominate the cluster solution. Furthermore, it enables the user to identify the importance of each item in the cluster solution [49]. The classification variables were z transformed and the cluster analysis was performed using a predetermined fixed number of clusters (k = 3), aiming to uncover a pattern in the data set, i.e., a latent group within the CRPS-CLP spectrum.

The model fit was assessed by Schwarz’s Bayesian Information Criterion [50] and evaluated by the average silhouette coefficient, an internal validity index representing cluster cohesion and separation quality, ranging between 0 and 1; the closer to 1, the better the model [51]. 

A crosstab analysis was performed to test the association (χ2) and its strength (Cramer’s V-rc) between the derived clusters and the participants’ original diagnosis, and a MANOVA to detect differences in the classification variables based on cluster groups. 

The cluster sensitivity was measured using ANOVA and Kruskal–Wallis tests to explore differences in the CSS score, MPQ-SF, Bath-BPD, and pro-inflammatory plasma cytokines levels depending on the three cluster groups.

Lastly, a spearman correlation was conducted to test the correlations between the pro-inflammatory cytokines and the pain measures -evoked and clinical pain (MPQ-SF). Bonferroni/Mann–Whitney analyses were used to detect significant mean differences using a pairwise comparison method. The Bonferroni correction was conducted in cases of multiple comparisons.

#### Missing Variables

The thermal and pain threshold tests were added as an additional descriptive measure of the cohort and were tested on a subsample of CRPS (n = 33) and CLP (n = 25) subjects. Twenty-eight CRPS and six CLP patients were enrolled before the protocol was established and therefore were not examined by these measures. The pro-inflammatory cytokine samples were collected from a subsample of CRPS (n = 20) and CLP (n = 13) subjects. Only measures with less than 10% missing values were included in the cluster model; the completion of the data, if required, was based on mean imputation [52].

### 2.7. The Study Protocol

All subjects were diagnosed according to the clinical Budapest Criteria and underwent the CSS evaluation by a pain specialist physician. Each participant completed four or five one-hour research sessions in a quiet room. The sessions included (1) blood samples performed during daylight hours between 8:00 AM to 2:00 PM, conducted by a paramedic or a physician; (2) psychophysical tests including thermal and pain thresholds, evoked pain measures, and allodynia area in percentages; and (3) self-administered questionnaires including demographic, psychological, and clinical pain, performed by a trained occupational therapist. The research sessions were conducted in random order, except for the first meeting, which was dedicated to acquainting the participants with the research team and completing the demographic questionnaires.

## 3. Results

### 3.1. Patients

The socio-demographics, pain characteristics, and comorbid medical diagnostic data of the CRPS and CLP participants are shown in Table 1. There were no significant differences between the groups regarding age, years of education, and disease duration (*p* > 0.05). In the CRPS group, the female/male ratio was 32/29 (52.5/47.5%). In the CLP group, the ratio was 23/8 (74.2/25.8%), with significantly more females than males (*p* = 0.044, Φ = 0.210). The average 24-h pain level and the current pain level based on the CSS index were significantly higher in the CRPS group (*p* < 0.001).

Medications and drug usage: the medical pain therapy included opiates, antiepileptics, anti-depressives, sleep drugs, analgesics, and B-phosphonate medications. Six participants suffered from hypertension, and two from hypothyroidism and used relevant medications. In addition, 16 participants had a medical license for cannabis. During the research period, the participants did not report substantial adverse drug reactions [53]; however, a complete medical follow-up regarding this issue was impossible as most of the participants were not fully hospitalized.

### 3.2. Thermal and Pain Thresholds Profile

**Figure 1:**
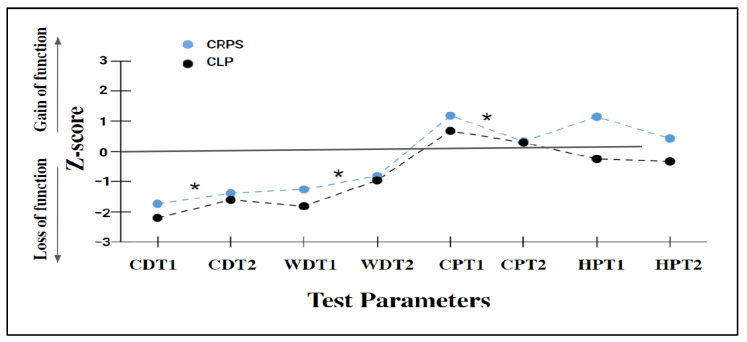
The thermal and pain threshold profile. Note. CRPS—Complex Regional Pain Syndrome; CLP—Chronic Limb Pain; CDT1—Cold Detection Threshold in the involved limb; CDT2—Cold Detection Threshold in the non-involved limb; WDT1—Warm Detection Threshold in the involved limb; WDT2—Warm Detection Threshold in the non-involved limb; CPT1—Cold Pain Threshold in the involved limb; CPT2—Cold Pain Threshold in the non-involved limb; HPT1—Heat Pain Threshold in the involved limb; HPT2—Heat Pain Threshold in the non-involved limb. Gain of function (z > 1); ‘loss of function’ (z < −1). Pathologic gain of function (z > 1.96) pathologic loss of function (z < −1.96). * *p* < 0.05- significant differences between the affected and non-affected sides in each group.

Statistically significant effects of the tested side were found in all the thermal tests (*p* < 0.05), except for HPT (*p* > 0.05). Specifically, the involved limb showed more sensitivity to painful stimuli (gain of function) and less sensitivity to non-painful stimuli (loss of function). Although the differences between the groups were insignificant, the CLP group showed more loss of function for CDT and WDT and the CRPS showed more gain of function for CPT and HPT. The differences between the affected and non-affected sides were more prominent in the CLP than in the CRPS group. The data are shown in Table 2 and in Figure 1.

### 3.3. The Cluster Analysis Model

The derived model yielded three clusters group (CRPS, CLP, and Mixed), The ‘CRPS’ group included 78.7% CRPS patients and 6.5% CLP patients; the ‘CLP’ group included 64.5% CLP patients and 4.9% CRPS patients; and the ‘Mixed’ included 16.4% CRPS and 29% CLP patients. Each cluster group comprised both pain-evoked and psychological measures and therefore the cluster was named CRPSycho-noci. χ2 analysis showed a significant correlation between the cluster and the original diagnosis (Φ = 0.741, *p* < 0.001). 

**Figure 2:**
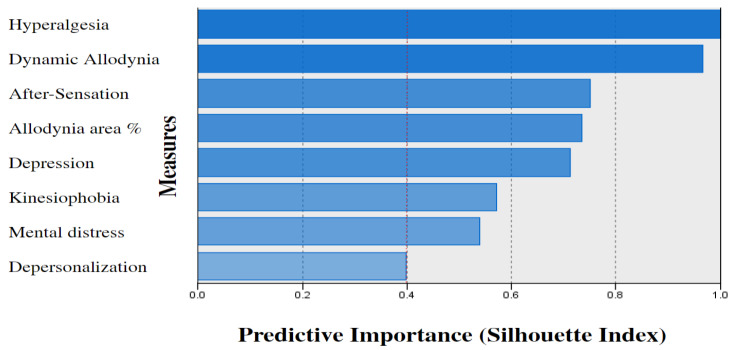
The measures’ predictive importance. Note: Two-step cluster analysis predictive importance measures. All measures are within the cutoff level of 0.4 silhouette index.

The cluster quality was fair (silhouette = 0.4); a silhouette coefficient equal to or above 0.50 indicates a good model fit [45]. The cluster cohesion indexes were good, with all measures having a silhouette value range between 0.4 and 1. The most important predictors were hyperalgesia and mechanical allodynia indicating a perfect model fit (silhouette = 1). The silhouette value of each measure (i.e., predictive importance) is shown in Figure 2.

**Figure 3:**
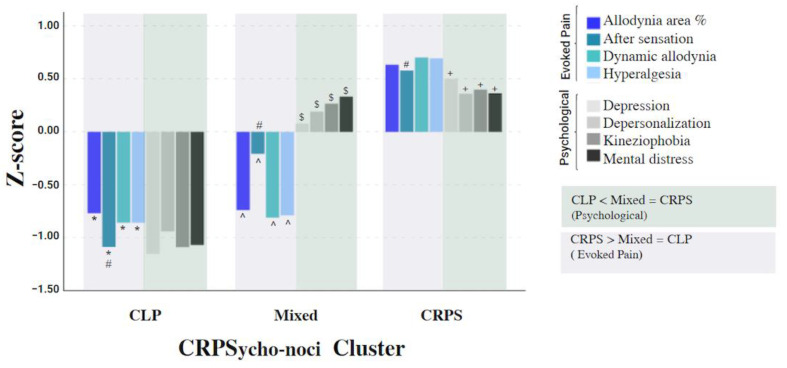
The three groups of the CRPSyco-noci cluster. Note. CRPS—Complex Regional Pain Syndrome; CLP—chronic limb pain; evoked pain and psychological measures distinguish between CLP, Mixed, and CRPS groups. All measures were z transformed. CLP showed lower values than CRPS in evoked pain measures (* CLP vs. CRPS) and CRPS showed higher values than CLP in psychological measures (+ CRPS vs. CLP). The Mixed group showed lower scores than CRPS in evoked pain measures (^ Mixed vs. CRPS) and higher scores than CLP in psychological measures ($ Mixed vs. CLP). After-sensation significantly differed in all groups (#).

The differences in the intensity of the measures between the cluster groups are shown in Table 3. The MANOVA model was significant (*F* (16, 164) = 18.94, *p*<0.001; Wilk’s Λ = 0.12, η² = 0.64), dynamic allodynia and hyperalgesia demonstrated the largest effect size (η² > 0.58). A post-hoc analysis revealed significant differences between the CRPS and CLP groups in all measures: the ‘CRPS’ demonstrated higher scores for psychological and evoked pain vs. the ‘CLP’. The ‘Mixed’ group exhibited similarities to CRPS in psychological measures and to CLP in evoked pain measures. The data are presented in Figure 3.

### 3.4. The Model Sensitivity

The secondary analysis aimed to test the model’s sensitivity in detecting differences between the cluster groups regarding the CSS score, clinical pain (MPQ-SF), Bath-BPD, and pro-inflammatory cytokines. The results are shown in Table 4. The analysis revealed significant differences in CSS scores between all the cluster groups (*p* < 0.001). The post-hoc analysis revealed that the CRPS, Mixed, and CLP cluster groups demonstrated the highest, moderate, and lowest scores, respectively. In addition, the CRPS group showed significantly higher clinical pain intensity, Bath-BPD scores, and TNF-α serum levels vs. the CLP group. However, the Mixed group did not differ significantly from the CRPS group. IL-6 serum levels did not differ significantly between the cluster groups.

### 3.5. Correlation between the Pro-Inflammatory Cytokines and the Pain Measures

The correlations of TNF-α and IL-6 serum levels with the evoked and clinical pain measures of hyperalgesia, dynamic allodynia, allodynia area, after-sensation, and MPQ-SF were tested by Spearman correlations, separately for each cytokine. After a Bonferroni correction, the adjusted alpha level was 0.01 (α = 0.05/5). The correlations that remained significant were between TNF-α and MPQ-SF (*r*s = 0.51, *p* = 0.002, *n* = 33) and between TNF-α and allodynia area (*r*s = 0.44, *p* = 0.001, *n* = 33).

## 4. Discussion

The study aimed to identify the clinical phenotypes of subgroups along the CLP-CRPS spectrum.

We used a unique classification method based on evoked pain and psychological measures that revealed a cluster solution based on a combination of these two realms (named the CRPSyco-noci cluster). The cluster comprised three groups: CRPS, CLP, and Mixed, which highly correlated with the original diagnoses and differed significantly in their CSS means. The most predictive factors, which shaped the cluster cohesion and separation, were mechanical sensitivity and pain after-sensation, with the CRPS group showing a higher pain hypersensitivity and psychological distress. 

Interestingly, the Mixed group, as named, showed a mixed pattern; it was similar to the CRPS group in the psychological measures and to the CLP group in evoked pain measures. Further testing of the cluster sensitivity revealed that the CSS score was significantly different between CRPS, Mixed, and CLP cluster groups demonstrating high, moderate, and lowest scores, respectively. Likewise, the CRPS group demonstrated significantly higher clinical pain, TNF-α serum level, and Bath-BPD score than the CLP, with no differences in IL-6 serum level. Nonetheless, these parameters did not differentiate CRPS from the Mixed group. On the contrary, the CRPS group showed a combination of gain and loss of thermal sensory function, and the CLP showed a more dominant loss of sensory function but these differences were insignificant. These findings further emphasize the role of central sensitization as a mechanism that differentiates between the chronic limb pain disorders tested here. 

### 4.1. Central Sensitization Processes That Differ between the Cluster Groups 

The study results show that the most discriminating between-group measures were mechanical hyperalgesia and dynamic allodynia. Both are clinical manifestations of the central sensitization process that occurs due to increased excitability and synaptic efficacy of neurons in the ascending transmitting pathways [54]. Furthermore, in the cases where the tests were undergone in the secondary hyperalgesia area, these serve as an additional indication of central sensitization [55]. The results indicate that the CRPS participants were more influenced than the CLP subjects by these central processes, demonstrating possible nociplastic pain. As we did not collect data regarding the participants’ comorbidity, such as cognitive problems, sleep disturbances, fatigue, or other senses hypersensitivity, we use the term ‘possible’ as recommended by the IASP clinical criteria of nociplastic pain [21,56], which is characterized as a pro-nociceptive profile [57]. This profile may be derived from a neural hyper-responsiveness in the ascending transmitting pathways that causes enhanced facilitatory processes at the spinal and supra-spinal levels, and/or reduced neural activity in the inhibitory pathways interrupting the endogenous analgesia. In both situations, this results in pain amplification [57,58]. 

A few potential mechanisms may explain the pro-nociceptive profile in CRPS: (i) Higher post-injury pain level (i.e., in the week after the trauma) [59,60]; the CRPS group showed higher clinical and evoked pain levels than the CLP group, although it is unknown whether their pain levels shortly after the trauma were also higher. Yet, this group was diagnosed with CRPS due to continuous and disproportionate pain levels, which is one of the CRPS diagnosis criteria [1,4,61]. Moreover, ongoing nociceptive pain is a risk factor for developing nociplastic pain [21] and continuous high pain intensity can exhaust the capacity of participants’ ability to inhibit pain due to their need to cope with the ongoing pain in the long-term [57,58]. (ii) Another potential mechanism is the inflammatory process, which is derived from and interacts with central sensitization processes [13,62]. Inflammatory processes are suggested to play a substantial role in the pathogenesis of CRPS, both in acute and chronic stages, producing sensitization via secretion of pro-inflammatory cytokines, e.g., TNF-α and IL-6 [13,62,63]. Indeed, the CRPS group showed a higher TNF-α serum level, implying its role in the inflammatory process at the chronic CRPS stages. (iii) Predisposing genetic factors causing heightened pain sensitivity and reduced efficiency of pain inhibition processes can contribute to the pro-nociceptive profile [55,57,64]. This possibility was supported by a previous study that suggested a pre-existing inter-individual difference in the pain modulatory network activity among CRPS patients [55]. This could not be tested here because this was a cross-sectional study. On the contrary, we found no group differences in thermal thresholds, which is in line with another previous study that demonstrated no differences in thermal sensitivity between CRPS and CLP patients when comparing both body sides [22]. The thermal thresholds evaluate the function of the peripheral nervous system, namely C and Aδ fibers [65], which may be similar in the CRPS and CLP groups in our cohort. This further supports central mechanisms as a diagnostic marker. 

Mechanical pain sensitivity measures were found to differentiate the CRPS from the CLP group [3,22], but interestingly, pain after-sensation was the only measure that differed significantly between the three cluster groups. After-sensation is an index of central sensitization [54,66] reflecting sensitization of second-order neurons. Namely, wide dynamic range pain transmission neurons that produce prolonged pain after discharge, which outlasts the period of stimulation [67]. In a case where hyperalgesia is identified, this response can also occur after a non-painful sensory stimulus, as was seen in the current study [68]. These results align with a previous study that demonstrated after-sensation and secondary hyperalgesia, both manifestations of central sensitization, as a dominant underlying mechanism in nociplastic pain and specifically in chronic CRPS [69]. 

### 4.2. Psychological State Differences between the Cluster Groups

The most important discriminative measure was depression, which is mutually associated with chronic pain via shared brain mechanisms [70]. For example, the nigra-subthalamic circuit is involved in the maintenance of both hyperalgesia and depression and is modulated by the impairment of substantia nigra reticulata-subthalamic nucleus GABAergic projection [71]. In CRPS, depression is a measure associated with pain severity [26,72,73] and disability [26], reflecting these prominent aspects of the disease burden [74]. Kinesiophobia was the second most important measure among the psychological measures. Kinesiophobia reflects a fear of movement and (re)injury [75], manifested as pain-related fear and avoidance behavior [76]. A systematic review among chronic musculoskeletal pain patients revealed that a greater degree of kinesiophobia is associated with greater levels of pain intensity, pain severity, disability, and a lower quality of life [77]. In CRPS, fear of pain is a predictor of pain intensity [26] and disability [75]. Furthermore, kinesiophobia is correlated with longer symptom durations and a lower illness perception [78]. These may lead to the adoption of poor coping strategies including immobilization, protecting, and neglecting the limb [78], which themselves aggravate the severity of the syndrome. Accordingly, this implies that there is a link between kinesiophobia and other psychological measures that establishes a vicious cycle of pain, emotional distress, and functional limitation in CRPS. 

### 4.3. The Mutual Association between Pain Hypersensitivity and Psychological State

The cluster classification, with each group characterized by various degrees of pain hypersensitivity and psychological distress, suggests a mutual association between pain and emotional suffering [79]. The suggested underlying central processes are brain circuits that are involved in pain chronicity, e.g., the cortico-limbic pain circuit including the prefrontal cortex, anterior cingulate cortex, amygdala, and nucleus accumbens. All these brain areas are involved in the emotional and affective processing of pain as well as in its modulation, amplification, and chronicity [80,81]. fMRI assessment of CRPS patients has shown that hyperalgesia and allodynia stimulation in the affected side produced an increase and widespread brain activation in somatosensory regions, prefrontal cortex, bilateral insula, and anterior cingulate cortex compared to the non-involved side [82,83]. The two latter brain areas are involved in interoception, inherently integrated pain, emotion, and body awareness [84]. Thus, the mutual association between pain and psychological distress in CRPS and CLP represents their bidirectional influence at the chronic pain stages yet, with a different relative contribution depending on the magnitude of the central sensitization [85,86,87,88].

The Mixed group showed similarity to the CRPS group in their psychological profile along with low evoked-pain intensity, like the CLP group. This pattern implies that although the Mixed group displayed a relatively minor clinical presentation of CRPS signs and symptoms, there was substantial psychological distress, specifically a high intensity of kinesiophobia and somatization. Moreover, the CSS scores demonstrated significant differences between groups with severe, moderate, and mild scores in the CRPS, Mixed, and CLP groups, respectively. These findings further validate CSS as an outcome measure [4] and support the cluster sensitivity in detecting different clinical phenotypes reflecting different chronic pain states. Examining the Mixed group distribution revealed that it comprised 29% of the CLP and 16.4% of the CRPS group, suggesting that this group can fit CRPS not otherwise specified, CRPS with remission of some features subtypes [17], or mild CRPS patients.

### 4.4. TNF-α and Central Neuro-Inflammation Processes

Our cluster model was further tested for TNF-α and IL-6 serum levels. TNF-α was found to be higher in the CRPS and Mixed groups compared to CLP, while the differences in IL-6 levels between the cluster groups were insignificant. TNF-α is a pro-algesic cytokine released by microglia and astrocytes, is involved in the central neuro-inflammation process [89], and serves as a neuromodulator in the spinal cord dorsal horn. It enhances synaptic plasticity after peripheral injury through the excitation of synaptic transmission. In CRPS, central sensitization processes are more prominent due to ongoing pain and continuous nociceptive input [63,90]. This process leads to the ongoing activation of glial cells in the dorsal horn and the brain [91], which in turn increases the release of pro-inflammatory cytokines and enhances central sensitization [62]. As a result, these central processes contribute to pain persistency in CRPS [13,92]. The CRPS literature regarding the role of IL-6 and TNF-α in the chronic stages is controversial and based on substantial variability in patient characteristics (i.e., acute vs. chronic), testing methods (i.e., CSF, blister fluid, mRNA, and blood), and type of control group (healthy vs. limb pain or the contralateral noninvolved limb) [93]. Thus, it is difficult to draw clear conclusions. However, a systematic meta-analysis has demonstrated an elevated level of TNF-α in a serum sample of chronic CRPS, while IL-6 was found to be elevated in a blister fluid only [94]. In the current study, positive correlations were found between TNF-α, clinical pain intensity, and allodynia area but not IL-6. This further confirms the potential involvement of TNF-α as a marker of centralized pain and neuro-inflammation processes in chronic CRPS. 

### 4.5. BPD in CRPS

Testing the cluster sensitivity in detecting differences in the Bath-BPD score revealed a significantly higher Bath-BPD score in CRPS compared to CLP, with no differences between the Mixed and CRPS groups. BPD is a well-known phenomenon reported in CRPS [59] and CLP [95], yet to a lesser extent in the latter [96,97]. BPD is proposed to be a consequence of learned nonuse processes derived from pain, movement suppression, and fear avoidance in CRPS patients [98]. Although learned nonuse processes can also result from movement avoidance as a pain preventive strategy in the CLP group, we can postulate that this process was amplified in the CRPS group due to mechanical hypersensitivity (i.e., allodynia and hyperalgesia), leading to touch avoidance as well. 

Kuttikat et al. [99], suggested two potential neurocognitive mechanisms for somatosensory misperception that leads to BPD in CRPS. The first mechanism is derived from a disruption in the quality of ascending sensory input arising from the involved limb. This disruption is due to neuro-inflammatory processes leading to greater weight on brain prediction processes without online sensory precision, resulting in limb misperception and BPD. The second mechanism is derived from decreased attention to the involved limb, which may be due to psychological distress (e.g., fear of pain, depression) leading to ‘cognitive neglect’ and limb depersonalization. Our results suggest that these mechanisms probably exist to a greater extent in CRPS compared to CLP since we found significantly greater BPD in the CRPS group. 

### 4.6. Limitations

The study has a few limitations: (i) Although we used the thermal tests as bilateral measures, the evoked pain measures (i.e., the intensity of allodynia, hyperalgesia, and allodynia area) were not tested bilaterally; hence, we could not evaluate the central sensitization process in the contralateral side by using these measures. (ii) The study did not include the conditioned pain modulation test. This test could have gained further understanding of the differences in the pain inhibition modulation function between groups. (iii) The pro-inflammatory samples were taken from a sub-sample that included 33 subjects, thus limiting the strength of the results, which will need further validation in future studies. (iv) A few subjects used cannabis which has possible anti-inflammatory effects on TNF-α levels. However, as the IL-6 levels did not differ between groups, we do not think it was an influential factor. 

## 5. Conclusions

The research findings show that central processes underlying nociplastic pain can distinguish between different CLP conditions. CRPS and its possible subtypes have a unique combination of psychological and pain phenotypes derived from central sensitization and central neuro-inflammation processes. These processes possibly lead to changes in the perception of nociceptive stimuli resulting in pain amplification and mechanical hypersensitivity, along with changes in cognitive and affective brain areas, which may lead to psychological distress and BPD. Future studies can use the described classification method to identify specific markers in other chronic primary pain syndromes, and therefore promote the planning of multimodal, personalized pain medicine interventions.

## Figures and Tables

**Table 1:** Demographic data in CRPS and CLP patients.

Variables	CRPS	CLP	*p*
Patients, n	61	31	
Age (years), mean (SD)	34.69 (11.52)	39.0 (15.09)	0.164
Sex (female/male), n (%)	32/29 (52.5/47.5)	23/8 (74.2/25.8)	0.044
Education (years), mean (SD)	12.93 (2.14)	13.71 (2.25)	0.111
Disease duration (days), mean (SD)	728.62 (903.96)	775.81 (843.25)	0.809
Work status, n (%)			
Working	15 (24.59)	13 (41.93)	
Not working	43 (70.49)	14 (45.16)	
Soldier	3 (4.91)	4 (12.90)	
Type of injury, n (%):			
Fracture	31(50.81)	14(45.16)	
Trauma	27 (44.26)	15 (48.38)	
Other	3 (4.91)	2 (6.45)	
Limb involved, n (%):HandLeg			
	24 (39.35)	12 (38.7)	
	37 (60.65)	19 (61.29)	
Side involved, n (%):			
Rt.	31 (50.81)	12 (38.71)	
Lt.	30 (49.19)	19 (61.29)	
Nerve injury, n (%)	38 (62.35)	16 (51.61)	
Pain characteristics, mean (SD):			
NRPS—Current	7.15 (1.5)	4.29 (2.59)	<0.001
NRPS—24 H	7.42 (1.38)	5.11 (2.57)	<0.001
Comorbid diagnosis n (%):			
Cardiovascular/Hematology	5 (8.19)	3 (9.66)	
Endocrine diseases	5 (8.19)	1 (3.22)	
Enzyme deficiency/Allergies	4 (6.55)	1 (3.22)	
Neurodevelopmental disorder	3 (4.91)	2 (6.45)	
Missing	1 (1.63)	6 (19.35)	

Note. CRPS—Complex Regional Pain Syndrome; CLP—Chronic Limb Pain; Other—inflammation, acute disease, spontaneous onset. The presence of nerve injury was determined by a pain specialist physician based on clinical tests and/or EMG findings.

**Table 2:** The differences in thermal and pain threshold between limbs in CRPS and CLP groups.

Variables, Mean (SD)	CRPS (n = 33)		CLP (n = 25)		*F* (1, 56)	*p*	η^2^
	Involved	Not involved	Involved	Not involved			
CDT	−1.74 (1.87)	−1.39 (1.45)	−2.21 (1.26)	−1.61 (1.19)	4.08	0.048	0.068
WDT	−1.26 (1.82)	−0.82 (1.55)	−1.82 (1.18)	−0.96 (1.35)	7.27	0.009	0.115
CPT	1.19 (1.16)	0.33 (1.21)	0.68 (1.18)	0.29 (1.19)	10.74	0.002	0.161
HPT	1.15 (2.11)	0.43 (1.61)	−0.02 (1.60)	0−.03 (1.30)	1.55	ns	

Note. CRPS—Complex Regional Pain Syndrome; CLP—Chronic Limb Pain; CDT—Cold Detection Threshold; WDT—Warm Detection Threshold; CPT—Cold Pain Threshold; HPT—Heat Pain Threshold.

**Table 3:** Differences in the intensities of the measures between the cluster groups.

Variables, Mean (SD)	Cluster ‘CRPS’ (*n* = 50)	Cluster ‘Mixed’ (*n* = 19)	Cluster ‘CLP’ (*n* = 23)	*F* (2, 89)	Multiple pairwise comparisons	η^2^
Dynamic allodynia	7.32 (1.53)	2.11 (2.92)	1.94 (2.78)	64.96 *	CLP = Mixed < CRPS	0.59
Hyperalgesia	7.72 (2.58)	2.86 (2.99)	2.63 (2.38)	61.67 *	CLP = Mixed < CRPS	0.58
After-sensation	7.66 (1.39)	5.11 (3.47)	2.23 (2.85)	42.97 *	CRPS > Mixed > CLP	0.49
Allodynia area %	5.84 (3.03)	0.97 (1.54)	0.87 (2.11)	41.64 *	CLP = Mixed < CRPS	0.48
Depression	28.55 (10.06)	23.63 (7.11)	9.29 (5.42)	40.01 *	CRPS = Mixed > CLP	0.47
Kinesiophobia	42.89 (6.08)	41.79 (6.62)	30.43 (7.44)	29.89 *	CRPS = Mixed > CLP	0.40
Mental distress	2.64 (0.57)	2.62 (0.45)	1.72 (0.39)	27.81 *	CRPS = Mixed > CLP	0.38
De-personalization	76.61 (47.29)	68.31 (45.51)	12.66 (18.09)	19.21 *	CRPS = Mixed > CLP	0.30

Note. CRPS—Complex Regional Pain Syndrome; CLP—Chronic Limb Pain; Mixed—a mixed cluster group. * *p* < 0.001.

**Table 4:** Cluster group differences in CSS, MPQ-SF, Bath-BPD scores and pro-inflammatory cytokines.

**Variables, Mean (SD)**	**Cluster ‘CRPS’** **(*n* = 50)**	**Cluster ‘Mixed’** **(*n* = 19)**	**Cluster ‘CLP’** **(*n* = 23)**	***F* (2, 89)**	**Multiple pairwise** **comparisons**	**η^2^**
CSS Score	12.08 (2.54)	8.93 (2.83)	6.16 (3.28)	36.74 **	CRPS > Mixed > CLP	0.45
MPQ-SF	27.22 (9.01)	22.21 (9.63)	10.61 (7.41)	28.2 **	CRPS = Mixed > CLP	0.38
Bath-BPD	25.61 (10.07)	21.36 (7.17)	12.93 (10.54)	13.49 **	CRPS = Mixed > CLP	0.23
**Variables, Median** **(Interquartile range)**	**Cluster** **CRPS** **(*n* = 17)**	**Cluster** **Mixed** **(*n* = 7)**	**Cluster** **CLP** **(*n* = 9)**	***H* (2)**	**Multiple pairwise** **comparisons**	***r***
TNF-α (pg/mL)	1.27 (0.50)	1.77 (1.28)	0.61 (0.42)	6.28 *	CRPS = Mixed > CLP	-0.53
IL-6 (pg/mL)	1.97 (2.48)	1.39 (0.85)	3.73 (1.63)	4.15	ns	-0.30

Note. CRPS—Complex Regional Pain Syndrome; CLP—Chronic Limb Pain; Mixed—a mixed cluster group; CSS—CRPS Severity Score; MPQ-SF—McGill Pain Questionnaire Short Form; BATH-BPD—Bath Body Perception Disturbances questionnaire; ** *p* < 0.001; * *p* < 0.05.

## Data Availability

The data presented in this study are available on request from the corresponding author.
